# Investigation of antibacterial and wound healing activities of the extract of *Rhodotorula mucilaginosa* endophyte isolated from cucumber leaves

**DOI:** 10.1038/s41598-025-16234-8

**Published:** 2025-08-28

**Authors:** Duaa Eliwa, Maisra M. El-Bouseary, Mahmoud H. Farghali, Thanaa A. El-Masry, Amany E. Ragab

**Affiliations:** 1 Department of Pharmacognosy, Faculty of Pharmacy, Tanta University, Tanta, Egypt; 2 Department of Microbiology and Immunology, Faculty of Pharmacy, Tanta University, Tanta, Egypt; 3 Department of Pharmacology and Toxicology, Faculty of Pharmacy, Tanta University, Tanta, Egypt

**Keywords:** LC–MS/MS, *Cucumis sativus*, *R. mucilaginosa*, Biofilm, Endophytic fungi, Carotenoids, *Pseudomonas aeruginosa*, Antibacterial, Anti-inflammatory, Biofilms, Plant sciences

## Abstract

Endophytic fungi represent a reservoir of pharmacologically essential secondary metabolites. The current study focused on the antibacterial properties of the endophytic yeast-like fungus *Rhodotorula mucilaginosa* (*R. mucilaginosa*) isolated for the first time from *Cucumis sativus* (cucumber) leaves. After isolation, *R. mucilaginosa* was identified by 18S rRNA gene sequencing and was cultured on Asian rice for production of fungal metabolites. Then, its phytochemical profile was elucidated using LC–HRESI–MS/MS technique to reveal 22 compounds which are mainly carotenoids and fatty acids in nature. Dried ethyl acetate extract of *R. mucilaginosa* was tested for antimicrobial activity against *Pseudomonas aeruginosa* isolates. The extract of *R. mucilaginosa* (ERM) showed minimum inhibitory concentrations with a range from 64 to 512 µg/mL. The crystal violet assay was utilized to determine the effect of ERM on the tested isolates’ ability to produce biofilms. The percentage of strong biofilm-forming isolates dropped from 20 to 3.3% and 10%, respectively, after the treatment with ½ and ¼ MICs of ERM. When tested isolates of *P. aeruginosa* were exposed to sub-MICs of ERM, their cell size significantly decreased, and their biofilm matrix was reduced. The in vitro anti-inflammatory activity of ERM was supported by the marked decrease in TNF-α gene expression in the cells treated with either ERM or piroxicam compared to the control cells. The wound healing effect of ERM was investigated in vitro, where ERM showed improvement in the wound healing process. In addition, the wound healing effect of ERM was investigated in vivo using a model of infection in rats, where the wounds were infected with *P. aeruginosa*. The histological investigation of the wound showed a remarkable improvement in the group treated with ERM. Therefore, further investigation is required to explore the possible application of ERM as a potential antibacterial agent, which could aid in our fight against pathogenic microbes that have become resistant.

**Supplementary Information:**

The online version contains supplementary material available at 10.1038/s41598-025-16234-8.

## Introduction

Endophytes are ubiquitous microorganisms that live in the inner tissues of healthy plant parts without causing any disease-like symptoms^1^. Endophytes help plants grow either directly by increasing their growth or indirectly by inhibiting phytopathogens from growing^2^. Furthermore, endophytes can directly inhibit plant pathogens through antagonistic action, neutralize toxic elements in the environment, or indirectly promote induced plant systemic resistance to help suppress plant diseases^3^. It has been reported that endophytes may produce a variety of bioactive metabolites in a single plant such as terpenoids, xanthones, polyketides, steroids, anthraquinones, alkaloids, phenols, and isocoumarins^4,5^. Endophytic fungi are known for producing novel, beneficial bioactive compounds with valuable biological properties like antibacterial^6^, antifungal^7^, anti-inflammatory, anti-oxidant^8^, antiprotozoal^9^, antituberculosis^10^, immunomodulatory^11^ and antiproliferative activities^12^. This makes them a great source of medications for curing a wide range of diseases and has possible applications in the food, cosmetics, pharmaceutical, and agriculture industries^13^.

Antimicrobial resistance is on increasing, causing a “global emergency” and demanding the development of new strategies for fighting antibiotic-resistant microbes^14^. Endophytic secondary metabolites exhibit significant efficacy against a range of bacterial and fungal species known to cause illnesses in humans^15^. It has been proven that endophyte extracts exhibit antimicrobial activity against a variety of drug-resistant microbes, including *Shigella flexneri*, MRSA, MRSE, and *Bacillus* sp.^16^. These endophytes may contribute to the host plant’s health and defense and could serve as an alternative source of valuable metabolites, particularly for antimicrobial, antioxidant, or anticancer purposes. *Cucumis sativus*, commonly known as cucumber, is a widely cultivated plant belonging to the Cucurbitaceae family. Native to South Asia, particularly the Indian subcontinent, cucumber has been domesticated and grown across many parts of the world for thousands of years, both as a food crop and for its medicinal properties^17^. Traditionally consumed for its refreshing and hydrating qualities, the fruit is rich in water, vitamins, and minerals^18^. Beyond its culinary use, *C. sativus* holds a significant place in traditional medicine systems such as Ayurveda and Traditional Chinese Medicine. It has been employed for its anti-inflammatory, analgesic, antidiabetic, antioxidant, and antimicrobial activities^19^. Extracts from various parts of the plant, including fruit, seeds, and peels, have been used to treat conditions such as hyperglycemia, hypertension, constipation, and skin disorders^20^. These medicinal properties are believed to be due to a rich array of phytochemicals such as flavonoids, tannins, and phenolic acids. The ethnopharmacological significance of *C. sativus* suggests the presence of unique bioactive compounds, not only in the plant tissues but also potentially harbored within its endophytic microbial communities. Given its well-documented medicinal value and its potential as a reservoir of bioactive compounds, *C. sativus* represents a promising candidate for the isolation of endophytic microorganisms. These endophytes may contribute to or potentiate the plant’s therapeutic properties by producing secondary metabolites with pharmacological relevance. This rationale underpinned the selection of *C. sativus* as the source plant in the present study.

*Pseudomonas aeruginosa* is a Gram-negative opportunistic bacterial pathogen that is capable of causing a variety of human infections, including cystic fibrosis, otitis media, chronic obstructive pulmonary disorder, blood stream infections, and chronic wound infections^21,22^. *P. aeruginosa* is an organism that can produce an extensive list of virulence factors, promptly develop antibiotic resistance, and adapt to alterations in the environment^21^. The complex architecture of the biofilm of *P. aeruginosa* adds to its pathogenic potential by increasing its ability to evade immunity, causing persistent infections that are challenging to treat, and lead to treatment failure^23^. Moreover, pyocyanin, a redox-active secondary metabolite, is potentially accountable for the blue-greenish hue of *P. aeruginosa* colonies. Additionally, pyocyanin is harmful to human cells, as it damages host tissue, intensifies illness, and compromises the function of organs^24^.

Epidemiological research has demonstrated that *P. aeruginosa* infections can dramatically raise rates of death, morbidity, surgical intervention requirements, long-term medical care, and overall treatment costs^25,26^. Therefore, the current area of concern prioritizes effective treatment options for mitigating biofilm infections related to *P. aeruginosa.* Herein, the study aimed at investigating in vitro and in vivo antimicrobial activity of the yeast-like fungus endophyte *R. mucilaginosa* isolated from the leaves of cucumber against *P. aeruginosa*, and its extract was investigated in vitro for anti-inflammatory and wound healing effects.

## Results

### Identification of the isolated yeast-like fungus endophyte

**Fig. 1:**
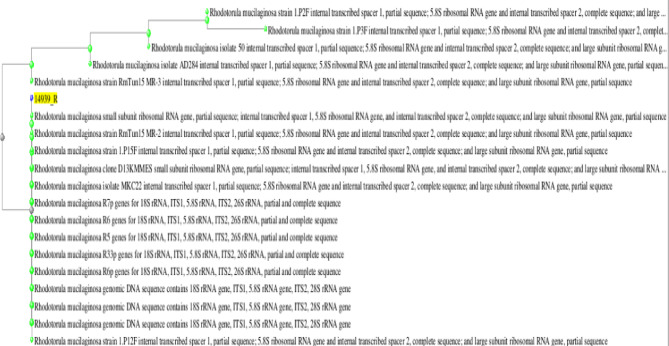
The isolated endophytic fungus’s phylogenetic tree based on the 18S rRNA gene sequence. The location of the isolated strain, *R. mucilaginosa*, is indicated by the yellow highlight.

Based on the 18S rRNA gene sequencing data and the macroscopic and microscopic characteristics of the isolated endophytic yeast-like fungus, this fungus was identified as a *R. mucilaginosa*, as demonstrated in Fig. 1. The accession number of the DNA sequence was LC497302.1 (Table 1).

**Table 1:** Identification data of *R. mucilaginosa* by 18S rRNA sequencing.

Identification	Highly similarity isolates	Highly similarity isolates Accession number	Identity %
*Rhodotorula mucilaginosa*	*R. mucilaginosa* strain isolate *C. sativus* leaf small subunit ribosomal 18S ribosomal RNA, partial and complete sequence	LC497302.1	96.06

### Phytochemical characterization of ERM

The ERM was analyzed utilizing HR-LCMS-QTOF technique. The compounds were tentatively identified based on their pseudomolecular ions m/z, MS/MS fragments, free databases search and published literature.

The results (Figure S1, Table 2) revealed 22 compounds which are mainly carotenoids and fatty acids in nature.

**Table 2:** HR-LCMS-QTOF characterization results of ERM.

NO	tR min	[M-H]^-^ (m/z)	MS/MS (m/z)	Identification
1	2.22	387.11700	341.10938, 179.05601	Caffeoyl glucoside
2	2.47	239.00189	195.04974, 111.00913	9,10-epoxy-octadecadidecenoic acid
3	3.01	405.03027	191.02002, 111.00894	4,4’-Diapo-phytofluene
4	14.72	227.09073	185.11786, 57.03557	Myristic acid
5	20.68	359.0675	233.10171, 75.00845	Lariciresinol
6	22.89	242.94417	174.95616, 112.9804	Biotin
7	23.29	174.95599	130.96590	2-Isopropyl malic acid
8	24.32	596.84583	435.91360, 342.12261, 248.43480, 112.98757	Tetra-hydroxy-tetrahydro-lycopene
9	25.11	242.94417	174.95576	2-Hydroxy-tetradecanoic acid
10	25.31	799.35853	556.06637, 456.17874, 362.65167, 112.98662	Derivative of tetrahydroxy-dihydro-lycopene
11	25.51	242.94417	174.95586	Isomer of 2-hydroxy-tetradecanoic acid
12	25.64	664.80231	556.00389, 449.19195, 343.13784, 233.10291, 112.98528	Derivative of tetrahydroxy-dihydro-lycopene
13	26.17	600.8244	495.27206, 233.10261, 83.05261	Tetrahydroxy-dihydro-lycopene
14	26.54	664.80231	555.99804, 449.20838, 334.58643, 233.10077, 83.05124	Derivative of tetrahydroxy-dihydro-lycopene
15	30.96	605.16583	437.12506, 293.17368, 112.98585	Tetrahydroxy-hexahydro lycopene
16	31.16	491.1273	424.28590, 377.12433, 226.95719, 112.98543	β- Carotene derivative
17	34.73	536.28369	491.26566, 412.98376, 239.12083, 112.98526	β- Carotene
18	35.21	534.14697	293.18544, 112.98692	Torulene
19	39.23	293.17662	236.10526, 71.01457	Hydroxy linolenic acid
20	44.98	353.21246	230.99812, 112.98662	Tricosanoic acid
21	46.39	339.2106	293.17936, 163.11042, 59.00677	Docosanoic acid
22	56.52	564.50987	421.01021, 281.24924, 144.92064	Torularhodin

### In vitro antibacterial activity

**Fig. 2:**
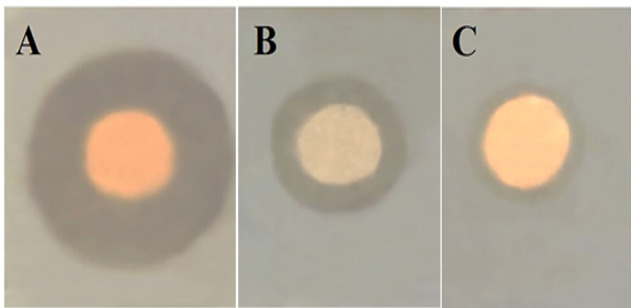
The potential antibacterial activity of ERM against *P. aeruginosa* (ATCC 27853) reference strain. ERM (**A**), gentamicin (**B**), and ethyl acetate (**C**) showed inhibition zone diameters (IZDs) of 25 mm, 16 mm, and 12 mm, respectively.

Using the agar-disc diffusion method, ERM demonstrated antibacterial activity against the reference strain of *P. aeruginosa* (ATCC 27853) (Fig. 2). Using the broth microdilution assay, we detected the MIC values of ERM against *P. aeruginosa* clinical isolates (n = 30). The range of reported MIC values was from 64 to 512 µg/mL (Table 3).

**Table 3:** MIC values of ERM against the tested *P. aeruginosa* isolates (n = 30).

MIC (µg/mL)	Number of isolates (%)
64	2 (6.7)
128	7 (23.3)
256	13 (43.3)
512	8 (26.7)

### In vitro anti-biofilm activity

The total number of biofilm producers *P. aeruginosa* isolates reduced from 28 to 21, 23, and 26 following the treatment with ½, ¼, and ⅛ MICs of ERM, respectively (See supplementary Table S1). The treatment of ½ and ¼ MICs of ERM resulted in a reduction of the percentage of strong biofilm-forming isolates from 20% to 3.3% and 10%, respectively (Table 4).

**Table 4:** Effect of the treatment by ERM (½ and ¼ MICs) on the biofilm formation.

Categories of Biofilm Production	Number of isolates (%)			
	Pre-treatment	Post-treatment		
		⅛ MIC	¼ MIC	½ MIC
None	2 (6.7)	4 (13.3)	7 (23.3)	9 (30)
Weak	9 (30)	8 (26.7)	6 (20)	5 (16.7)
Moderate	13 (43.3)	13 (43.3)	14 (46.7)	15 (50)
Strong	6 (20)	5 (16.7)	3 (10)	1 (3.3)
Total producers	28 (93.3)	26 (86.7)	23 (76.7)	21 (70)

### Bacterial morphological changes induced by ERM

**Fig. 3:**
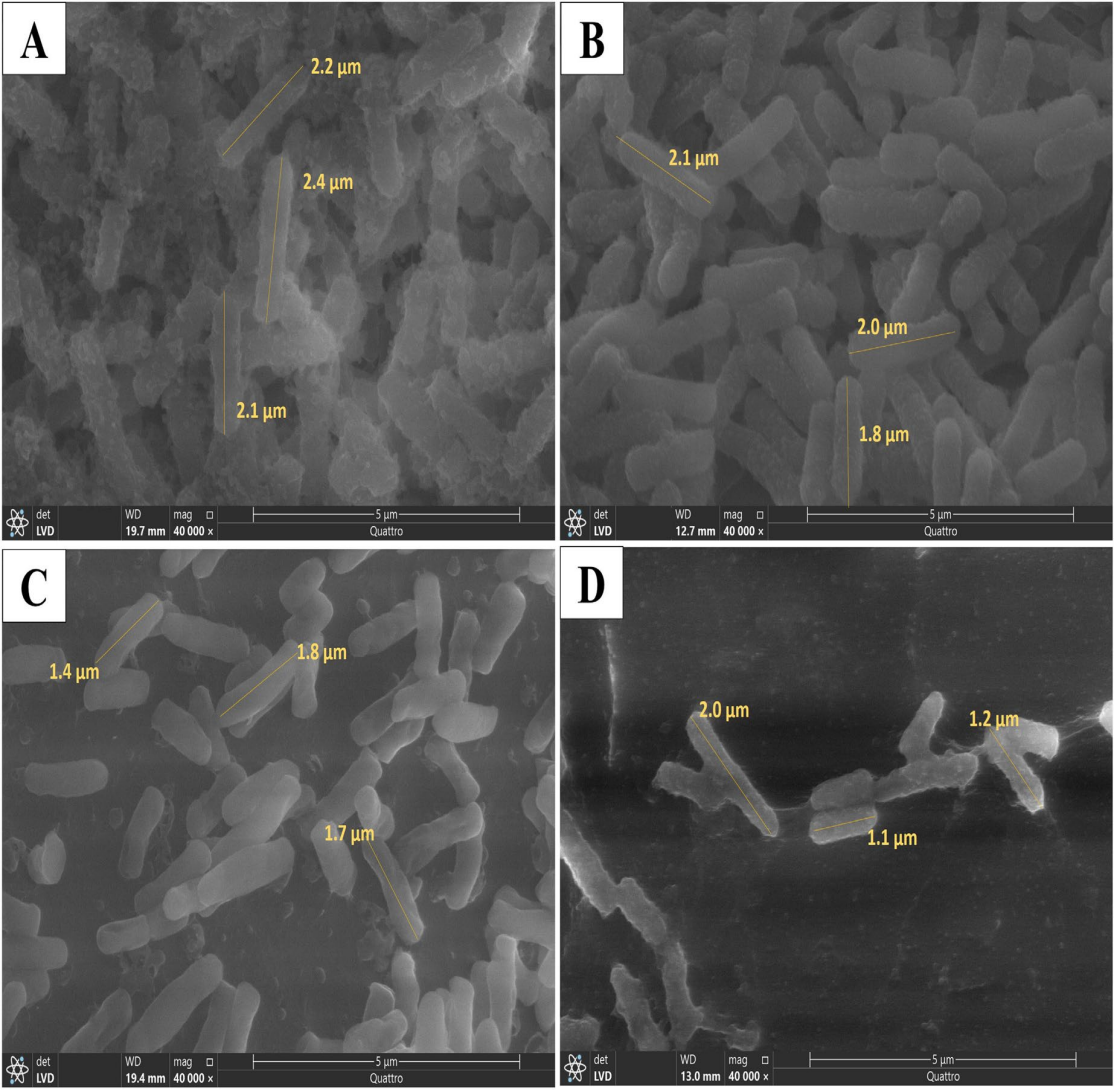
Scanning electron micrograph of *P. aeruginosa* (P22) untreated cells (control) (**A**) and the treated cells with ERM at ⅛ MIC (**B**), ¼ MIC (**C**), and ½ MIC (**D**).

**Fig. 4:**
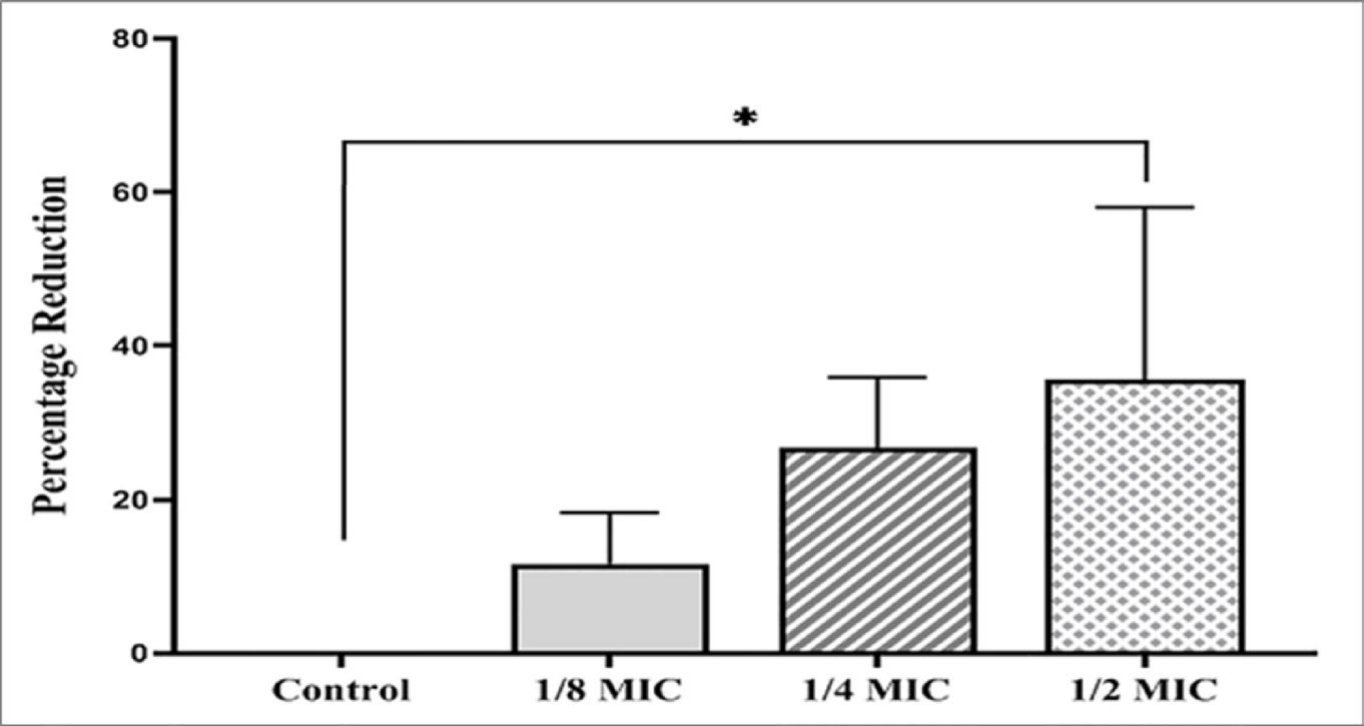
The percentage reduction of *P. aeruginosa* (P22) cell length following the treatment with different sub-MICs of ERM. *p value < 0.05, ANOVA test.

Exposing *P. aeruginosa* (P22) to sub-MIC of ERM causes signifigant reduction in cell size and decrease the biofilm matrix formed by tested isolates (Fig. 3). The treatment of bacterial cells with ½ MIC of ERM showed a significant reduction (36% ± 17.2) in the bacterial cell length (*P* < 0.05) (Fig. 4).

### In vitro anti-inflammatory activity of ERM

**Fig. 5:**
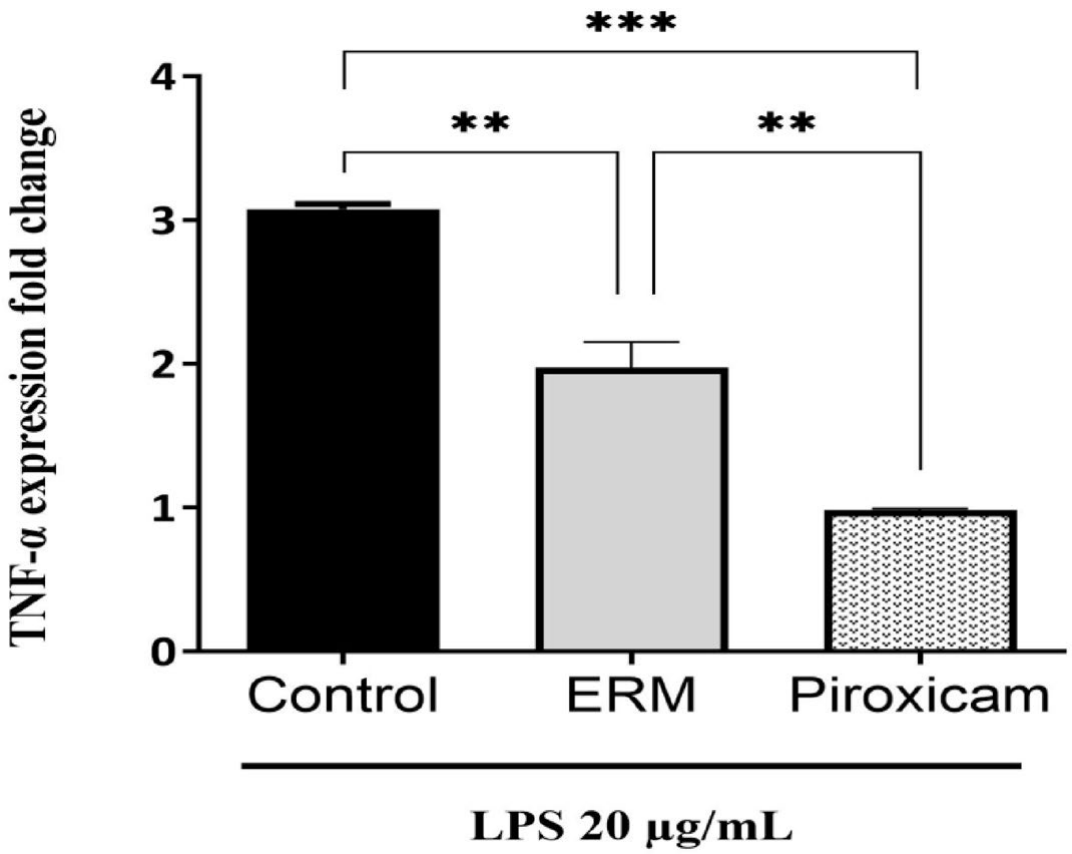
In vitro anti-inflammatory activity of ERM. Data represented mean ± standard deviation (n = 2), **p value < 0.01, ***p value < 0.001, ANOVA test.

Cytotoxicity of ERM was determined on WI38 human fibroblast cells, and IC_50_ of ERM was 23.62 µg/mL. The anti-inflammatory activity of one tenth of IC_50_ of ERM was tested on LPS-stimulated WI38 cells, in comparison with 10 µg/mL piroxicam. As shown in Fig. 5, LPS stimulation resulted in an increase in TNF-α gene expression (~ 3.08-fold ± 0.03). On the other hand, LPS-stimulated cells treated with ERM showed only ~ 1.98-fold increase ± 0.18, with a significant decrease in TNF-α gene expression compared to LPS-stimulated control cells (*p* = 0.004), while treatment with piroxicam resulted in ~ 0.98-fold change ± 0.01, with a significant reduction in TNF-α gene expression compared to LPS-stimulated control cells (*p* = 0.0006).

### ERM enhances the in vitro wound healing process

**Fig. 6:**
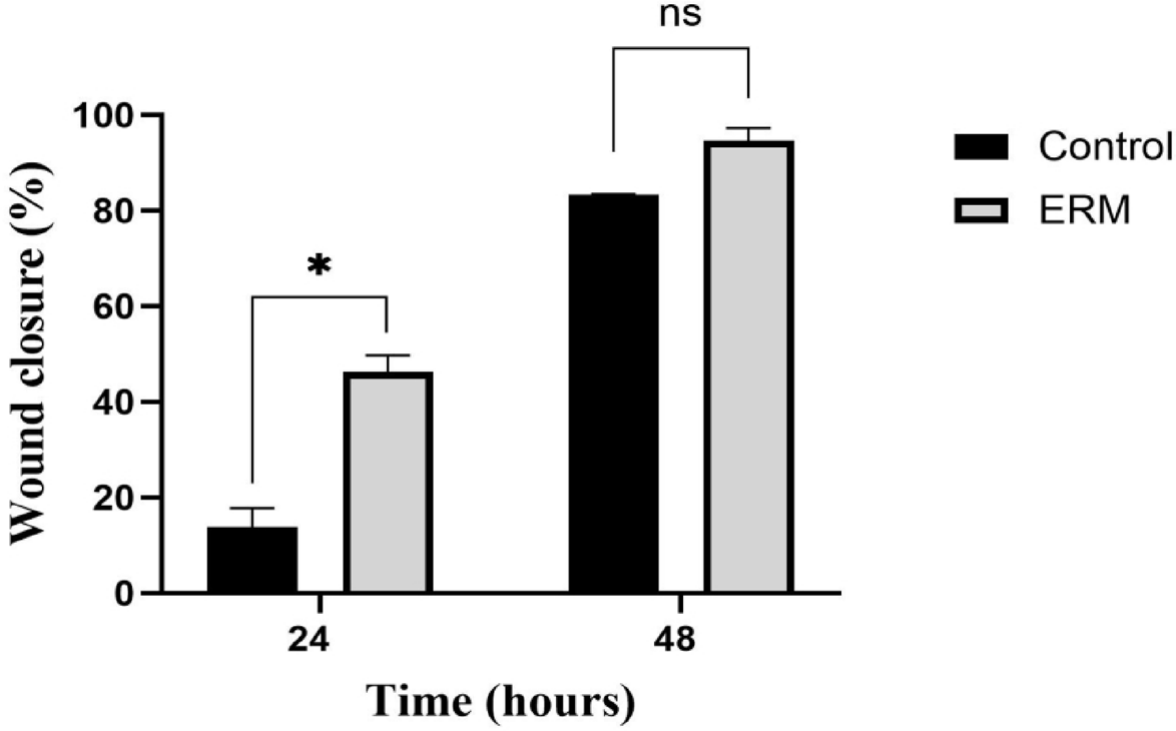
ERM enhances the in vitro wound healing process. Treatment with ERM increased the percentage wound closure, with statistical significance at 24 h. *p value < 0.05, two-tailed unpaired *t*-test.

Wound healing assay results revealed that ERM enhanced the wound healing process in WI38 human fibroblast cells (Supplementary Figure S2). The relative wound size in control and ERM-treated cells was quantified 24 and 48 h after wound induction. Treatment with ERM increased the percentage wound closure (46.28% ± 3.43 at 24 h and 94.66% ± 2.64 at 48 h) compared to the control cells (13.79% ± 3.98 at 24 h and 83.37% ± 0.05 at 48 h), with statistical significance at 24 h (*P* < 0.05) (Fig. 6).

### In vivo wound infection model

**Fig. 7:**
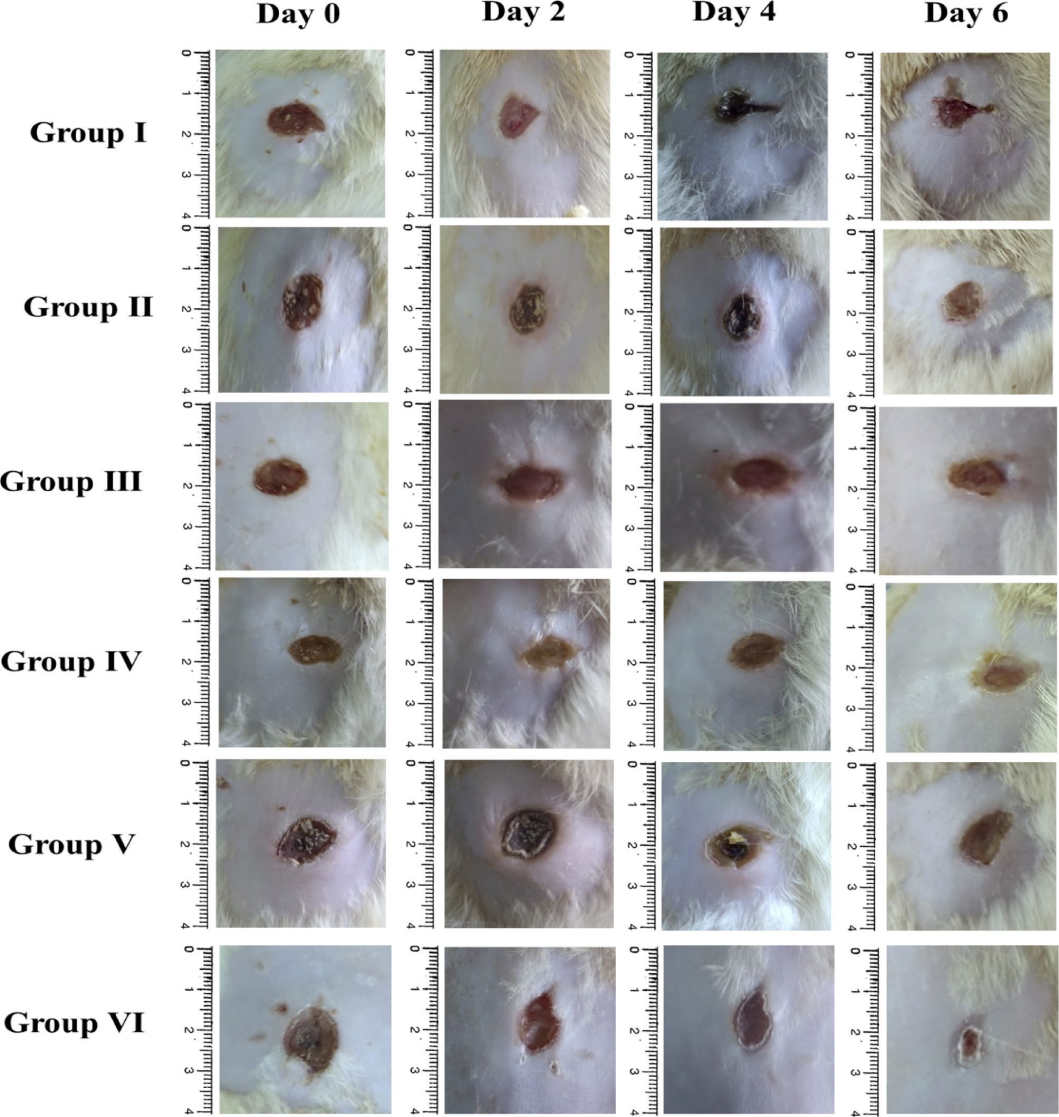
Macroscopic wound contractions were visualized in different animal groups at days 0, 2, 4, and 6.

The macroscopic healing rates of the animals under study are displayed in Fig. 7. The group under study had their wound surface size measured on days 0, 2, 4, and 6, respectively. On day six, wound scabs were seen in groups II-and V (treated with ERM) and VI (treated with gentamicin). ERM improved healing in uninfected wounds (Group II) relative to saline alone (Group I), reducing the wound area to 21.5% versus 35.8%, respectively, highlighting its anti-inflammatory or regenerative properties. Moreover, the infected groups treated with ERM (Groups IV and V) showed markedly better wound closure compared to the infected untreated control (Group III). On Day 6, the wound area was reduced to 20.2% in Group V (ERM 500 µg/mL) and 31.2% in Group IV (ERM 1000 µg/mL), compared to 57.1% in Group III. While gentamicin (Group VI) exhibited the most pronounced healing effect, with a wound area of 11.7% and an 84.5% reduction from baseline, the outcome observed in Group V was comparable, highlighting ERM’s potential as a natural therapeutic alternative. Notably, ERM at 500 µg/mL resulted in faster healing than the higher dose (1000 µg/mL), indicating a possible dose-dependent optimization.

**Fig. 8:**
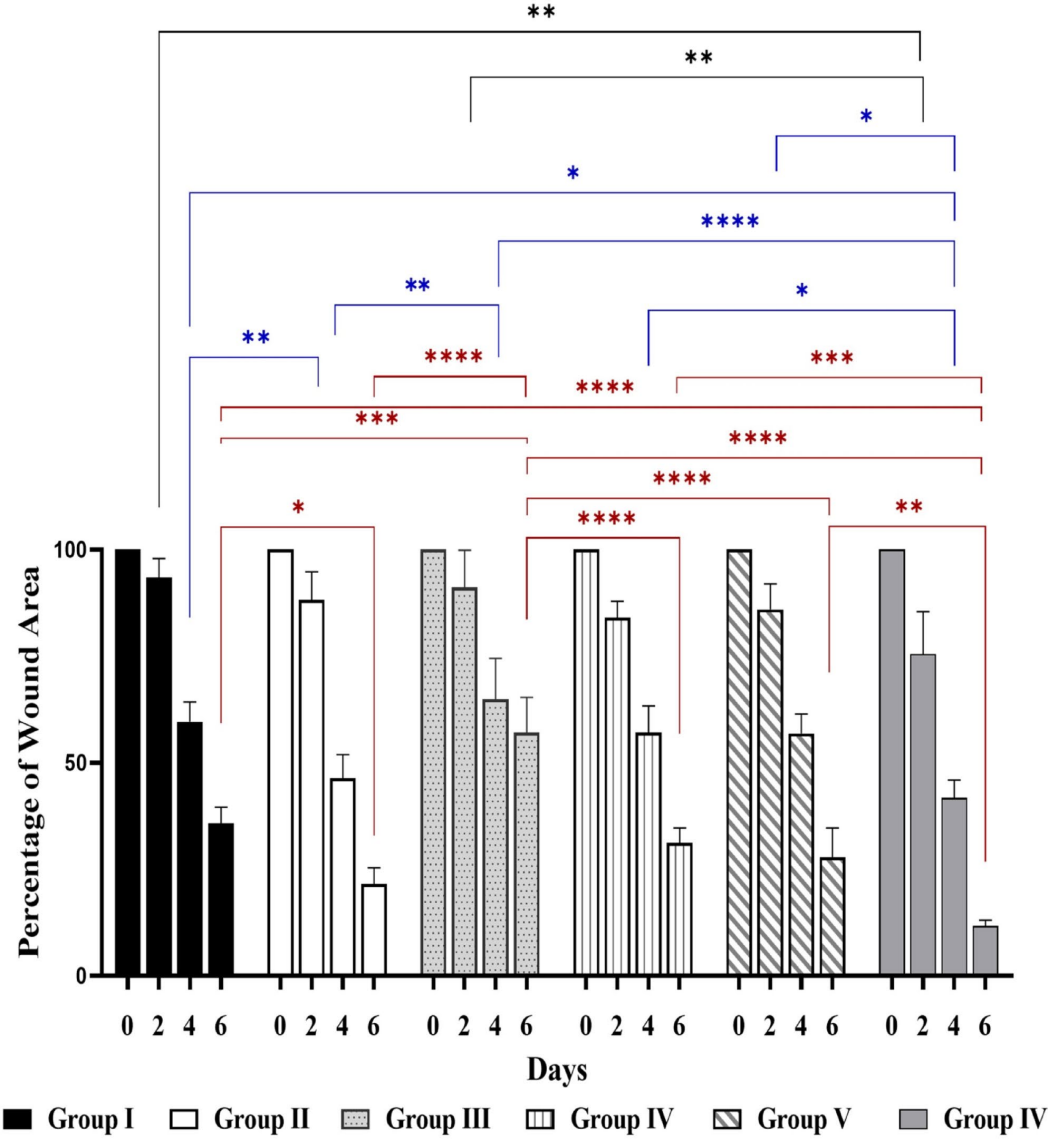
The percentage of wound area for a single rat was calculated trice as a representative in different animal groups at days 0, 2, 4, and 6. *p value < 0.05, **p value < 0.01, ***p value < 0.001, ****p value < 0.0001, ANOVA test.

As shown in Fig. 8, the percentage of wound area can be observed comparatively. More specifically, on day 6, groups IV, V (both treated with ERM), and IV (treated with gentamicin) showed comparable wound area percentages 28.3%, 20.2%, and 10.3%, respectively, compared to group III 47.9% (conrol wound infected), with significant reductions in the wound surface since both wounds were almost recovered.

**Fig. 9:**
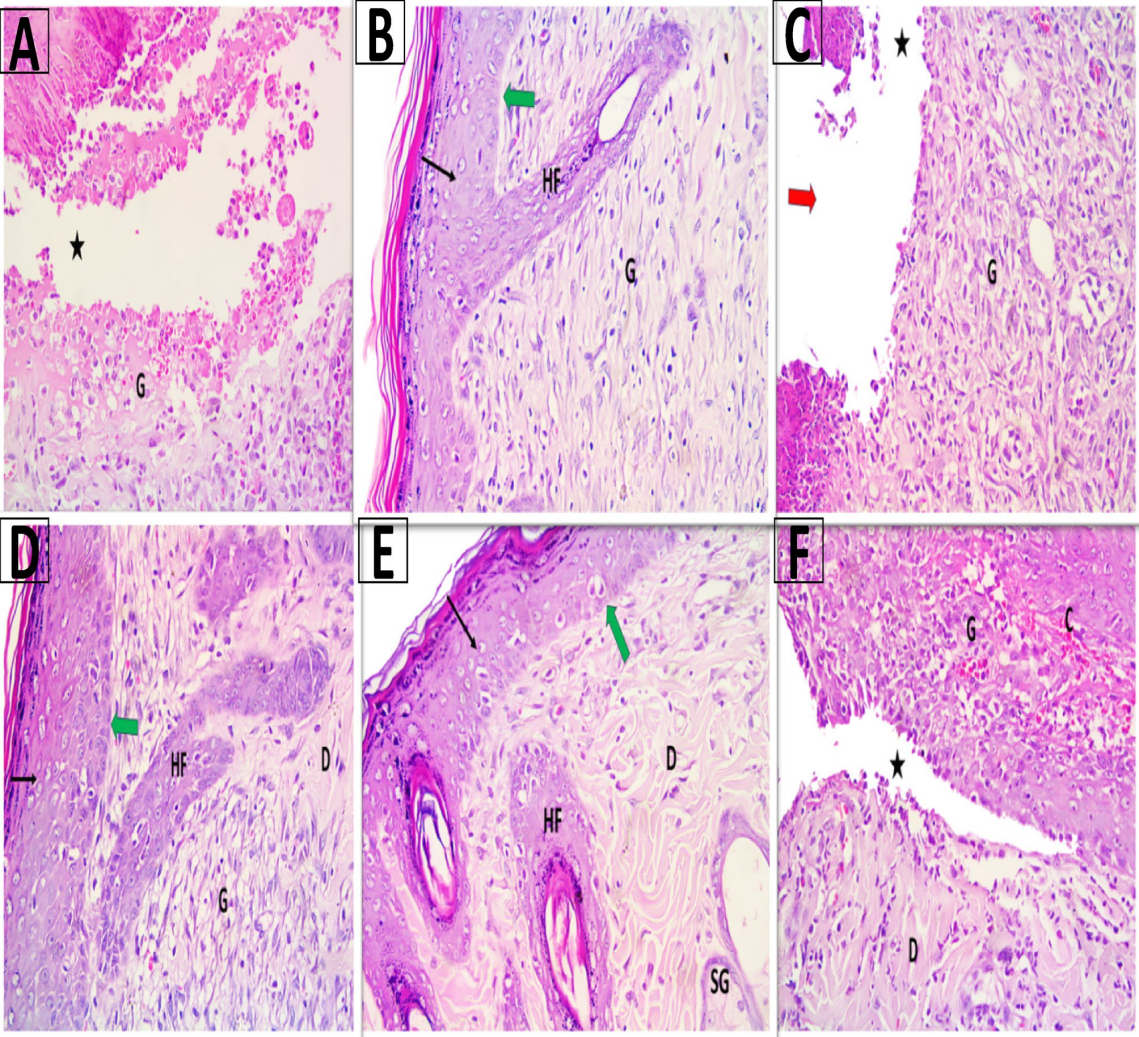
A photomicrograph of skin showing (**A**) Group I showed marked separation of epidermis from underlying connective tissue, granulation tissue and absence of skin appendages at the site of wound. (**B**) group II showed complete re-epithelialization of epidermis, intact basement membrane, granulation tissue and beginning of appearance of hair follicle. (**C**) Group III revealed disruption of epidermis, separation of epidermis from underlying connective tissue and granulation tissue. (**D**) Group IV showed separation of epidermis from underlying connective tissue, granulation tissue, organized connective tissue of dermis in most areas and absent skin appendages. (**E**) Group V showed complete re-epithelialization of epidermis, intact basement membrane, granulation tissue, organized connective tissue of dermis and appearance of hair follicle. (**F**) Group VI revealed complete re-epithelialization of epidermis, intact basement membrane, complete organization of connective tissue of dermis, appearance of multiple hair follicles and sebaceous glands. The disruption of epidermis (red arrow), re-epithelialization of epidermis (thin arrow), intact basement membrane (green arrow), separation of epidermis from underlying connective tissue (*), organization of connective tissue of dermis (D), granulation tissue (G), congestion (C), hair follicle (HF), sebaceous gland (SG). (H&E Mic. Mag. × 400). Groups III, IV, V, and VI were infected with *P. aeruginosa* (P22) isolate.

The histological evaluation of wound healing among different treated animal groups by hematoxylin and eosin (H & E) is presented in Fig. 9. The results demonstrated that ERM at 500 µg/mL (Group V) improved wound healing in infected tissues. It led to full re-epithelialization structured dermal layers, and the reappearance of hair follicles. These results were comparable to gentamicin (Group VI), which achieved the most complete tissue repair, including multiple hair follicles and sebaceous glands. ERM at 1000 µg/mL (Group IV) gave moderate results, suggesting a possible dose-dependent effect, with the lower concentration yielding more favorable healing outcomes. ERM (Group II) also encouraged re-epithelialization and early development of skin structures in non-infected wounds. This shows it might help tissues heal not just because of its antimicrobial effect but also due to its anti-inflammatory properties. On the other hand, the infected untreated group (Group III) had a damaged epidermis and loose connective tissue emphasizing how infection worsens healing and how ERM could help treat infected wounds.

## Discussion

A variety of secondary metabolites are known to be produced by endophytes and the majority of the compounds exhibit antimicrobial activities. It is expected that such properties include defense against numerous pathogens such as bacteria, viruses, fungus, nematodes, etc. for the host plant^27^. Recently, a red yeast-like fungus called *R. mucilaginosa* has shown promise for application in the industry^15^.

In this investigation, *R. mucilaginosa* was isolated from the leaves of cucumber as an endophyte. The phytochemical investigation of the ethyl acetate extract of this endophyte assured its identification. *Rodotorula* species are characterized by the carotenoid cotent especially torulene, torularhodin, β-carotene and lycopene derivatives^28–30^. The carotenoids in the *R. mucilaginosa* extract exhibited strong antioxidant activity. Furthermore, a significant antibacterial activity of carotenoids against antibiotic resistant bacteria (ARB) which could potentially have biological applications in food, cosmetic and/or hygiene supplements industries^31^.

Previous studies reported the antibacterial activity of fungal endophytes against variable bacterial species^15,32,33^. The current study focused on the antibacterial properties of the endophytic yeast-like fungus *R. mucilaginosa* isolated for the first time from *C. sativus* (cucumber) leaves. In the current study, the dried ethyl acetate extract of *R. mucilaginosa* derived from *C. sativus* leaves showed a potential in vitro antibacterial activity against *P. aeruginosa*, which was investigated by the agar-disc diffusion method as shown in Fig. 2 for a reference strain (ATCC 27853) and by 96-well plate for 30 isolates, the reduction of biofilm formation capability as indicated by the crystal violet assay (Table 4 and supplementary Table S1) and by scanning electron microscopy (Fig. 3), and the reduction of bacterial cell length as shown in Figs. 3 and 4. In addition, we investigated the effect of ERM treatment in an in vivo wound infection model, and found a remarkable improvement of the healing process of wounds infected with *P. aeruginosa* (Figs. 7, 8, 9). In agreement with our findings, a previous study screened methanolic extracts of *R. mucilaginosa* strains isolated from different sources for the in vitro antibacterial activity against six pathogenic bacterial strains (*Serratia marcescens, Escherichia coli, Pseudomonas aeruginosa, Micrococcus luteus, Bacillus cereus* and *Staphylococcus aurous*), where a *R. mucilaginosa* strain isolated from molasses showed high antibacterial activity against all the tested pathogens^34^. The anti-pseudomonal activity of our ethyl acetate extract of *R. mucilaginosa* may be attributed to the carotenoids which represent a main component of the extract. Vidya P. et al. tested the antimicrobial activity of the pigments extracted from *R. mucilaginosa* isolated from the mangrove sediments of North Kerala, India, and reported that the carotenoid pigment was responsible for the inhibitory activity against *Staphylococcus aureus*^35^. Also, the antimicrobial action of the carotenoids obtained from the methanolic extraction of a strain of *R. mucilaginosa* isolated from soil collected in InCheon-city, South Korea was reported against antibiotic-resistant bacterial strains^29^. In addition, it was reported that carotenoids of *R. glutinis* given via the intraperitoneal route to mice provided protection against *P. aeruginosa*^36^.

Our results showed that ERM enhanced the in vitro wound healing process in WI38 human fibroblast cells as shown in Fig. 6 and Supplementary Figure S2. In agreement with our findings, a previous study reported that carotenoids produced from marine pigmented bacteria improved the wound healing process of human skin fibroblasts and oral epithelial cells^37^. Another study reported that topical application of the extract of astaxanthin, which is a carotenoid, enhanced the rate of wound healing of dermal wounds in mice^38^. The topical application of lycopene, one of the carotenoids identified in our extract, was reported to have a potential wound healing effect in diabetic rats^39^.

In the current study, the in vitro anti-inflammatory effect of ERM was assessed by investigating the gene expression level of TNF-α, a proinflammatory cytokine, in LPS-stimulated human fibroblast cells. We found that ERM treatment markedly reduced TNF-α expression level. This anti-inflammatory effect may be attributed to the carotenoids in our extract. Notably, carotenoids isolated from marine bacteria were reported to have an anti-inflammatory effect^37^. Also, previous studies reported the anti-inflammatory effect of secondary metabolites of endophytic fungi^15,40^. To the best of our knowledge, there are no previous studies that investigate the in vivo effects of R. mucilaginosa in a wound healing model using immunocompetent hosts, making our findings novel. Kang K. et al*.* reported that R. mucilaginosa enhanced the secretion of cytokines, including TNF-α, in an immunosuppressed mouse model^41^; however, the differences in host immune status and experimental design may account for the differing outcomes.

Although the ERM demonstrated strong antibacterial activity in vitro, this effect was not equally reflected in the in vivo results. The apparent variation between the strong antibacterial activity observed in vitro and the relatively modest effects in vivo is a well-recognized phenomenon in natural product research and antimicrobial testing. Several factors can account for this difference. In vivo systems are inherently more complex than in vitro models. The bioavailability, stability, and ability of natural products to reach the site of infection can be limited in vivo, which may reduce their effectiveness. Additionally, the host immune response and wound environment play a critical role in therapeutic outcomes. Interactions between the extract, the pathogen, and the host’s immune system, along with factors such as local inflammation, wound exudate, enzymatic breakdown, and immune cell activity, can influence or even mask the direct antibacterial effects observed under controlled in vitro conditions^42^.

As mentioned above, we reported the antibacterial, anti-inflammatory, and wound healing activities of the dried ethyl acetate extract of *R. mucilaginosa* isolated for the first time from *Cucumis sativus* (cucumber) leaves. However, further mechanistic studies are needed to exactly determine the active compounds responsible for these effects, the potential mechanism of wound healing, and other factors affecting the inflammation process. Identification of these compounds may provide new approaches used in the production of antimicrobials.

## Conclusion

The aim of the current investigation was to provide insights into the in vitro and in vivo antimicrobial action of crude extract from endophytic yeast-like fungi against *P. aeruginosa*. Our research showed that *R. mucilaginosa* crude extract exhibited a potent antimicrobial activity. These findings confirm that this endophytic yeast-like fungus provide a consistent supply of bioactive substances with a higher degree of inherent chemical variety. Purification and identification of these bioactive compounds, which may serve as a basis for developing of new antibacterial drugs to treat infectious disorders, are necessary for future research.

## Materials and methods

### Plant source of the endophytic yeast-like fungus *R. mucilaginosa*

Samples of fresh and healthy leaves of cucumber were acquired from a local farm at Tanta city, Al- Gharbia governorate, Egypt. It was identified by staff member of Botany Department, Faculty of Science, Tanta University, Tanta. A voucher specimen (PD-7-22-D3) has been preserved at Pharmacognosy Department at Tanta University, Tanta.

### Isolation and purification of the endophyte yeast-like fungus *R. mucilaginosa* from cucumber leaves

The cucumber leaf samples were rinsed with tap water until clean, sterilized by soaking in a solution of 70% ethanol for 60 s, rinsed 3–4 times using sterile water for ,and dried. A sterile dissection razor was then used to cut the leaves into 2 × 2 cm slices, which were then placed on agar plates with potato dextrose agar (PDA) medium supplemented with 250 mg/L amoxycillin to inhibit the growth of bacteria. The plates were incubated at room temperature for two weeks in order to ensure that the fungus had adequately grown. Growing fungus on agar plates was repeatedly inoculated with fresh PDA media resulting in pure strains of *R. mucilaginosa*.

### Molecular identification of the isolated yeast-like endophytic fungi

Following the manufacturer’s instructions, total fungal DNA was extracted and purified using the E.Z.N.A.® Fungal DNA Mini Kit (D3390-01, Omega BIO-TEK, USA). The isolated fungi were then characterized using the fungi universal ribosomal 18S rRNA gene. The forward primer was: 5′-TCCGTAGGTGAACCTGCGG-3′, and the reverse primer: was 5′-TCCTCCGCTTATTGATATGC-3′.

Gel documentation system (Geldoc-it, UVP, England), was applied for data analysis using Totallab analysis software, ww.totallab.com, (Ver.1.0.1). Aligned sequences were analyzed on NCBI website (http://www.ncbi.nlm.nih.gov/webcite) using BLAST to confirm their identity. Phylogenetic trees were designed and viewed through NCBI Tree Viewer (Tree Viewer 1.19.0, https://www.ncbi.nlm.nih.gov/tools/treeviewer/). The nucleotide sequences were also compared with *Rhodotorula* isolates sequences available in the GenBank.

### Preparation of the extract *of R. mucilaginosa*

The ethyl acetate extract of *R. mucilaginosa* (ERM) was prepared for subsequent characterization and biological testing. Under sterile conditions, a small block from the isolated fungus *R. mucilaginosa* was transferred to pre-autoclaved conical flasks containing Asian rice (100 g) in sterile water (ten flasks 1 L each containing 110 mL each). The fungus was grown under static conditions for four weeks at room temperature away from light. To determine the most suitable solvent for extracting bioactive metabolites from the fungal culture, preliminary solvent screening was performed using thin-layer chromatography (TLC). Several solvents of varying polarity, including dichloromethane, methanol, and ethyl acetate, were evaluated. Among the solvents tested, ethyl acetate showed the best metabolite profile on TLC, supporting its effectiveness in extracting a broad range of secondary metabolites. Ethyl acetate was used to extract the endophytic fungus three times before followed by filteration. The combined ethyl acetate extract was dried out using a rotary evaporator at 45 °C to obtain an ethyl acetate crude extract (120 gm, 12% yield, brown color).

### Phytochemical investigation of the ERM

Phytochemical analysis was conducted using the Agilent Technologies 6530 Q-TOF LC/MS, which was equipped with a Quat. Pump (G7104C), a Column Comp (G7116A), and an autosampler (G7129A). Two μL was the injection volume. The analytes were separated on a Zorbax RP-18 column from Agilent Technologies (150 mm × 3 mm, dp = 2.7μm), flow rate 3 mL/min. The solvent gradient consists of water containing 1% formic acid (A) and acetonitril containing 1% formic acid (B) as follows: 90% A for 0–2 min, linear gradient to 80% A for 2–10 min, linear gradiant to 20% A for 10–42 min, linear gradient to10% A for 42–53 min, linear gradiant to 100% B for 53–60 min. Using ESI in (-) ionization mode and a capillary voltage of 4500 V, mass spectra were obtained. The m/z range in which the mass spectra were recorded was 50–3000 m/z. The drying gas flow was 8 L·/min and the gas temperature was 200 °C. The collision energy was set at 10V, while the skimmer and fragmentator voltages were set at 65 and 130 V, respectively. The nebulization pressure was 58 psig.

### In vitro antibacterial activity

In the present study, for demonstration of the antibacterial action of ERM, thirty clinical MDR isolates of *P. aeruginosa* (P1-P30), were acquired from the culture collection of Microbiology and Immunology Department, Faculty of Pharmacy, Tanta University (see supplementary Table S1). The screening of the antibacterial activity of ERM against *P. aeruginosa* (ATCC 27853) reference strain was determined by agar disc diffusion technique. One of these discs was saturated with ERM (1000 µg/mL, dissolved in ethyl acetate), whereas the other discs were the positive and negative controls and contained gentamicin and ethyl acetate, respectively^43^. In 96-well micro-titration plate, the broth microdilution method was performed to determine the values of the minimum inhibitory concentration (MIC) of the tested isolates (n = 30)^44^.

### In vitro anti-biofilm activity

The ability of the tested isolates to produce biofilms and the impact of ERM (½, ¼, and ⅛ MICs) on this capacity were determined using the crystal violet assay, as previously reported^45^. Using a 96-well micro-titration plate, *P. aeruginosa* isolates were assigned to one of four categories based on their capacities for biofilm production according to the measured optical density (OD) values at 630 nm. The isolate was non-producer if OD ≤ ODc; weak producer if ODc < OD ≤ 2 × ODc; moderate producer if 2 × ODc < OD ≤ 4 × ODc; and strong producer if 4 × ODc < OD^46^. The percentage reduction in biofilm formation was determined as follows^47^:

$${\text{Percentage}}\,{\text{reduction}}\,{\text{in}}\,{\text{biofilm}}\,{\text{formation}} = \frac{{{\text{Control}}\,{\text{OD}}\,630\,{\text{nm}} - {\text{Treated}}\,{\text{OD}}\,630\,{\text{nm}}}}{{{\text{Control}}\,{\text{OD}}\,630\,{\text{nm}} }} \times 100$$

### Bacterial morphological changes induced by ERM

The effect of ERM on bacterial morphology was investigated using scanning electron microscopy (SEM). *P. aeruginosa* isolate (P22) was grown in a 6-well cell culture plate overnight at 37 °C in LB broth without and with ERM at different concentrations (½, ¼, and ⅛ MICs). Following three PBS washes of the wells (treated and control), the attached bacterial cells were fixed for two hours at ambient temperature using 2.5% glutaraldehyde in PBS buffer (pH 7.4) and then subsequently fixed for one hour at 4 °C using 1% OsO_4_ in PBS buffer (pH 7.4). A part of the bottom of the plate of each well was applied to the microscope slide, and ethanol was added, followed by air drying. Following their mounting on metal stubs and gold sputter coating, the slides were inspected under a scanning electron microscope (SEM) (Akashi Seisakusho, Japan)^48^.

### In vitro anti-inflammatory activity

First, piroxicam, a standard anti-inflammatory drug, and different concentrations of ERM dried extract were prepared in DMSO and suspended in serum-free RPMI medium. Cytotoxicity of ERM was determined on WI38 human fibroblast cells. In a 96-well plate, 3 × 10^3^ WI38 cells/ well were plated, then incubated with 10 µg/mL piroxicam and different ERM concentrations in CO_2_ incubator for 48 h (37 °C, 5% CO_2_, and 90% relative humidity). Then, the cells viability was determined by MTT assay^49^. Untreated cells served as the negative control, representing 100% cell viability. The anti-inflammatory activity of ERM was assessed by investigating the effect on the gene expression of TNF-α, a pro-inflammatory cytokine, in lipopolysaccharide (LPS)-stimulated WI38 cells^50,51^. In a 12-well plate, 5 × 10^4^ cells were plated with RPMI complete medium, then LPS (20 µg/mL) was added, and the plate was incubated for 24 h. After incubation, the plate was centrifuged for 5 min at 1650 rpm, and the supernatant was discarded. The cells were then incubated with 10 µg/mL piroxicam or 1/10 of IC_50_ of ERM for 48 h. After incubation, the plate was centrifuged, and RNA was isolated from the cells using RNA isolation kit (iNtRON Biotechnology, Korea) according to the manufacturer’s instruction. SensiFAST cDNA synthesis kit (Bioline, London, UK) was used to convert 1 µg RNA into cDNA. Gene amplification was conducted by qPCR using beta-actin as a housekeeping gene, where 1 µL of cDNA was mixed with 10 µL SensiFAST SYBR (Bioline, London), 0.5 µl of 10 pmol/mL forward primer and 0.5 µl of 10 pmol/mL reverse primer (Table 5). Nuclease-free water was used to complete the reaction volume to 20 µL. Using a CFX96™ Real-Time System (BIO-RAD, USA), the reaction mixture was heated at 95 °C for 10 min, followed by 40 cycles of 95 °C for 15 s, 60 °C for 30 s and 72 °C for 30 s. The cycle threshold (Ct) of TNF-α gene was normalized with Ct of the housekeeping gene.

**Table 5:** Sequences of TNF-α and beta-actin primers^56^.

Gene	Primer
TNF-α	F- CTCTTCTGCCTGCTGCACTTTG
	R- ATGGGCTACAGGCTTGTCACTC
Beta-actin	F- CACCATTGGCAATGAGCGGTTC
	R- AGGTCTTTGCGGATGTCCACGT

### In vitro wound healing assay

In vitro wound healing assay was conducted as previously described^52^, with modifications. In a 24-well plate, 10^4^ WI38 cells/ well were plated, then incubated in CO_2_ incubator for 24 h. After incubation, the culture medium was replaced with serum-free RPMI to wash the cell monolayer, then the cell monolayer was scratched (wounded) using a sterile 200 µL-pipette tip and washed with PBS to remove the cell debris. Then, the cells were incubated with or without 1/10 of IC_50_ of ERM for another 48 h. The migrating cells in the denuded zone were monitored and photographed using phase contrast microscopy. The Image J version 1.49 software was employed to quantify the relative wound size at 0, 24, and 48 h after wound induction.

## In vivo wound infection model

### Animals

Eighteen male Wistar albino rats were acquired from the animal house sited at the Faculty of Veterinary Medicine, Cairo University, Egypt. During a two-week period of acclimatization, the rats were fed standard pelleted food and pure water at 25 ± 2 °C with 12-h light/dark cycles. All standard procedures of handling of laboratory animals were followed according to ARRIVE guidelines. The in vivo experiment and protocol were endorsed by the research ethical committee of the Faculty of Pharmacy, Tanta University, Egypt (TP/RE/2/25 p-06).

#### Wound infection model

The rats were given a mixture of anesthesia via intraperitoneal injections of ketamine and xylazine (87.5 mg/kg and 12.5 mg/kg, respectively). Following that, a sharp blade was used to shave the backs of rats, and their dorsal skin was wiped with 70% alcohol. The skin biopsy punch was employed to induce approximately 10 mm full-thickness excisional skin incisions in the dorsal skin. The rats were divided randomly into six groups of three each (n = 3). The induction of wound infection was performed using 10 μL of the bacterial suspension (10^8^ CFU) of *P. aeruginosa* (P22) in all groups except group I and II, which represented the normal/negative control and non-infected treated group, respectively^53,54^. After 30 min of bacterial inoculation, groups I, II, III, IV, V and VI received 0.9% saline, ERM (1000 µg/mL), 0.9% saline, ERM (1000 µg/mL), ERM (500 µg/mL), and gentamicin (1 mg/mL), respectively. The treatment with different agents is based on saturation of the surface of the wound with each agent and waiting for adsorption to prevent leakage out of the skin surface. Surface swabs were collected from the skin of infected animals and cultured on cetrimide agar after 18 h of induction of the infection to confirm the colonization of the wound with *P. aeruginosa.*

Over the course of the experiment, the treatment that was administered on day 0 was repeated every day. In accordance with the American Veterinary Medical Association’s (AVMA) Guidelines for the Euthanasia of Animals (2020 Edition), rats were anesthetized on the sixth day of the experiment using isoflurane and then euthanized by cervical dislocation.

#### Macroscopic wound contractions

The day when the wounds were established was considered day 0. We observed the contractions of the wounds continuously on days 2, 4, and 6. The images of the wound were captured to follow up on the process of wound healing.

The wound sizes were determined using image analyzer software (Image J.2.0 software, USA). Wound area was presented as a percentage relative to the initial wound size according to the following equation:

$${\text{Percentage}}\,{\text{of}}\,{\text{wound}}\,{\text{area}} = \frac{{{\text{Wound}}\,{\text{size}}\,{\text{at}}\,{\text{the}}\,{\text{time}}\,{\text{of}}\,{\text{taking}}\,{\text{the}}\,{\text{image}}}}{{{\text{Initial}}\,{\text{wound}}\,{\text{size}}}} \times 100$$

#### Histological examination

After euthanasia, the entire wound, with a margin of approximately 10 mm of surrounding intact skin, was surgically removed for histological analysis. The collected tissues were preserved in 10% neutral formalin. Hematoxylin and eosin (H&E) were employed to stain the tissue after it was removed from formalin, placed in paraffin wax, and sectioned into five-micrometer blocks^55^.

### Statistical analysis

An ANOVA test was conducted with Prism version 9.1.0 (USA) to examine significance at *P* < 0.05. The data presented is the mean ± standard deviation. For the wound healing assay, an unpaired *t*-test was conducted to examine significance at *P* < 0.05, where the data represented the mean ± standard deviation.

## Supplementary Information

Below is the link to the electronic supplementary material.

Supplementary Material 1

## Data Availability

All data generated or analyzed during this study are included in this article and the supplementary file. Regarding the endophyte Accession number, it will be found in the following link (NCBI-Fungi): https://www.ncbi.nlm.nih.gov/nuccore/LC497302.
