# Supplementary material for: Investigation of antibacterial and wound healing activities of the extract of Rhodotorula mucilaginosa endophyte isolated from cucumber leaves
Source: Sci Rep. 2025 Aug 28;15:31730. doi: 10.1038/s41598-025-16234-8 (PMC12394409; doi:10.1038/s41598-025-16234-8)
Supplement: Supplementary file 1 — Supplementary Material 1 [file 41598_2025_16234_MOESM1_ESM.docx]

**Table S1.** ERM MIC values and the impact of ERM treatment on biofilm production by *P. aeruginosa* clinical isolates.

| **Isolate code** | **Resistance Pattern** | **ERM MIC (µg/mL)** | **Pre-treatment** | **Post-treatment** | | |
| --- | --- | --- | --- | --- | --- | --- |
|  |  |  |  | **⅛ MIC** | **¼ MIC** | **½ MIC** |
| P1 | AMK, CAZ, CIP | 128 | **W** | **N** | **N** | **N** |
| P2 | CIP, FEP, GEN | 256 | **M** | **W** | **W** | **W** |
| P3 | CAZ, CIP, IPM | 128 | **W** | **W** | **W** | **W** |
| P4 | GEN, IPM, LVX | 256 | **W** | **W** | **W** | **W** |
| P5 | IPM, LVX, TZP | 64 | **N** | **N** | **N** | **N** |
| P6 | AMK, CAZ, CIP, FEP | 128 | **M** | **M** | **M** | **M** |
| P7 | CAZ, CIP, FEP, GEN | 256 | **M** | **M** | **W** | **W** |
| P8 | AMK, CIP, IPM, MEM | 128 | **M** | **M** | **M** | **M** |
| P9 | CAZ, CIP, FEP, IMP | 512 | **S** | **S** | **S** | **M** |
| P10 | AMK, CIP, FEP, GEN | 256 | **W** | **W** | **N** | **N** |
| P11 | CIP, FEP, GEN, IPM | 128 | **M** | **M** | **M** | **M** |
| P12 | AMK, CAZ, CIP, IPM | 128 | **M** | **M** | **M** | **M** |
| P13 | AMK, CIP, FEP, IPM | 256 | **W** | **N** | **N** | **N** |
| P14 | FEP, GEN, IPM, LVX | 256 | **M** | **M** | **M** | **M** |
| P15 | CIP, GEN, IPM, MEM | 128 | **M** | **M** | **M** | **M** |
| P16 | AMK, FEP, IPM, LVX | 512 | **M** | **M** | **M** | **M** |
| P17 | AMK, IPM, LVX, TZP | 64 | **N** | **N** | **N** | **N** |
| P18 | AMK, CAZ, CIP, FEP, GEN | 512 | **M** | **M** | **M** | **M** |
| P19 | CAZ, CIP, FEP, IPM, MEM | 256 | **M** | **M** | **M** | **M** |
| P20 | CAZ, CIP, IPM, LVX, MEM, TZP | 512 | **S** | **S** | **S** | **M** |
| P21 | AMK, CIP, IPM, LVX, MEM, TZP | 256 | **W** | **W** | **W** | **W** |
| P22 | CAZ, CIP, FEP, IPM, LVX, MEM, TZP | 512 | **S** | **S** | **M** | **M** |
| P23 | CAZ, CIP, FEP, IPM, LVX, MEM, TZP | 512 | **S** | **S** | **S** | **S** |
| P24 | AMK, CAZ, CIP, FEP, IPM, LVX, MEM | 512 | **S** | **S** | **M** | **M** |
| P25 | AMK, CAZ, CIP, FEP, GEN, LVX, TZP | 512 | **S** | **M** | **M** | **N** |
| P26 | AMK, CAZ, CIP, FEP, GEN, LVX, TZP | 256 | **W** | **W** | **W** | **N** |
| P27 | CIP, FEP, GEN, IPM, LVX, MEM, TZP | 256 | **M** | **M** | **M** | **M** |
| P28 | AMK, CAZ, FEP, GEN, IPM, MEM, TZP | 256 | **W** | **W** | **N** | **N** |
| P29 | CAZ, CIP, FEP, GEN, IPM, LVX, MEM | 256 | **W** | **W** | **N** | **N** |
| P30 | AMK, CIP, CAZ, FEP, GEN, IPM, MEM | 256 | **M** | **M** | **M** | **M** |

N: None producer, W: Weak producer, M: Moderate producer, S: Strong producer. AMK: Amikacin, CAZ: Ceftazidime, CIP: Ciprofloxacin, FEP: Cefepime, GEN: Gentamicin IPM: Imipenem, LVX: Levofloxacin, MEM: Meropenem, TZP: Piperacillin-tazobactam


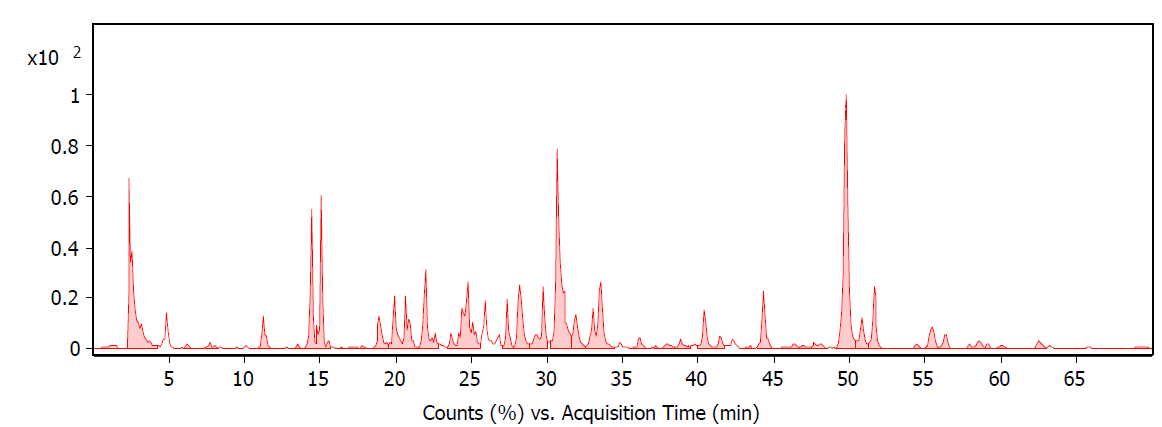


**Figure S1**: Total ion chromatogram for ERM as analyzed by HR-LCMS-QTOF


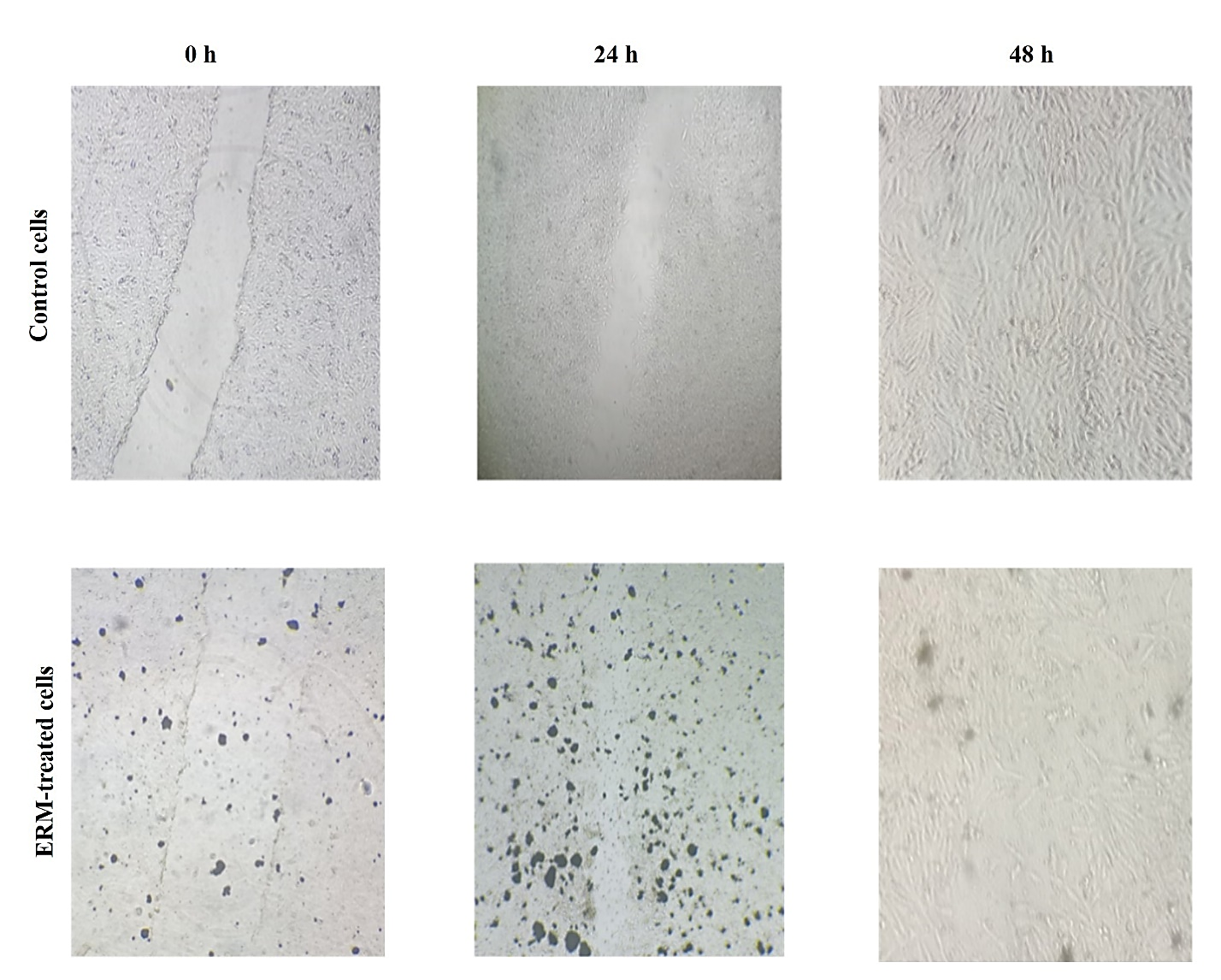


**Figure S2**. In-vitro wound healing effect monitored at 0, 24, and 48 h after wound induction at 40x magnification.
